# Medical Legislation in Georgia

**Published:** 1881-11

**Authors:** 


### MEDICAL LEGISLATION IN GEORGIA.

Elsewhere we publish, at the expense of considerable space, the three acts of the General Assembly of Georgia, passed at its recent session, which possess special interest for the medical profession.

We feel that the result achieved by the friends and advocates of these bills is a cause of congratulation, for, it may be truly affirmed that this is the first progressive medical legislation ever enacted in the history of the State, and it will, we believe, be gladly accepted as evidence of more liberal views, of a wiser statesmanship and of a juster appreciation of the needs of the people, on the part of those intrusted with the responsibility of making laws for the commonwealth, than has heretofore been exhibited.

Apart from the general popularity of the bills, both within and without the medical profession, perhaps the factor which contributed principally to success was the establishment, last winter, by the General Assembly, of a standing committee—in the House and in the Senate—on sanitation and hygiene, before whom all questions pertaining to sanitary matters must go.

Both committees, composed mainly or entirely of physicians, manifested great interest in the fate of these bills, and not only reported favorably upon them to their respective branches of the Legislature, without devision or dissent, but, individually, they became active champions of the cause and exerted a powerful personal, as well as official influence, in its behalf.

It has required systematic, patient and laborious work to accomplish what has been done, but now, that it is accomplished, we feel that it will be gratefully acknowledged by the friends of reform and progress throughout the State.

It is, for obvious reasons, desirable that the new laws should be viewed in their best light, and, even if they are not so rigid as some would have them, or if they vary in detail from the preferences or preconceived opinions of a few persons, we trust they will be accepted as the best that could be obtained, and that they will receive the cordial support of all classes, if not as a finality, at least, as a step in the right direction.

